# Regulation of NOXO1 Activity through Reversible Interactions with p22^phox^ and NOXA1

**DOI:** 10.1371/journal.pone.0010478

**Published:** 2010-05-04

**Authors:** Sujit Dutta, Katrin Rittinger

**Affiliations:** National Institute for Medical Research, Medical Research Council, London, United Kingdom; Griffith University, Australia

## Abstract

Reactive oxygen species (ROS) have been known for a long time to play important roles in host defense against microbial infections. In addition, it has become apparent that they also perform regulatory roles in signal transduction and cell proliferation. The source of these chemicals are members of the NOX family of NADPH oxidases that are found in a variety of tissues. NOX1, an NADPH oxidase homologue that is most abundantly expressed in colon epithelial cells, requires the regulatory subunits NOXO1 (NOX organizing protein 1) and NOXA1 (NOX activating protein 1), as well as the flavocytochrome component p22^phox^ for maximal activity. Unlike NOX2, the phagocytic NADPH oxidase whose activity is tightly repressed in the resting state, NOX1 produces superoxide constitutively at low levels. These levels can be further increased in a stimulus-dependent manner, yet the molecular details regulating this activity are not fully understood. Here we present the first quantitative characterization of the interactions made between the cytosolic regulators NOXO1 and NOXA1 and membrane-bound p22^phox^. Using isothermal titration calorimetry we show that the isolated tandem SH3 domains of NOXO1 bind to p22^phox^ with high affinity, most likely adopting a superSH3 domain conformation. In contrast, complex formation is severely inhibited in the presence of the C-terminal tail of NOXO1, suggesting that this region competes for binding to p22^phox^ and thereby contributes to the regulation of superoxide production. Furthermore, we provide data indicating that the molecular details of the interaction between NOXO1 and NOXA1 is significantly different from that between the homologous proteins of the phagocytic oxidase, suggesting that there are important functional differences between the two systems. Taken together, this study provides clear evidence that the assembly of the NOX1 oxidase complex can be regulated through reversible protein-protein interactions.

## Introduction

Superoxide is generated in a variety of tissues by NADPH oxidase (NOX) enzymes. It is an intermediate in the formation of reactive oxygen species (ROS), which have been implicated in a number of cellular functions including host defence, apoptosis, signalling and cell proliferation [1]–[7].

The first member of the NOX protein family to be identified was the phagocytic NADPH oxidase, whose gp91^phox^ subunit is now referred to as NOX2. This enzyme is primarily found in phagocytes where it plays a fundamental role in host defence against microbial infections [8]–[10]. This microbicidal activity was originally believed to be the main function of ROS. However, the recent discovery of a number of novel NOX family members in a diverse range of tissues, which include NOX1-5, Duox1 and Duox2, indicates that superoxide production by these proteins has a much wider physiological function [6], [7], [11]–[15].

**Figure 1:**
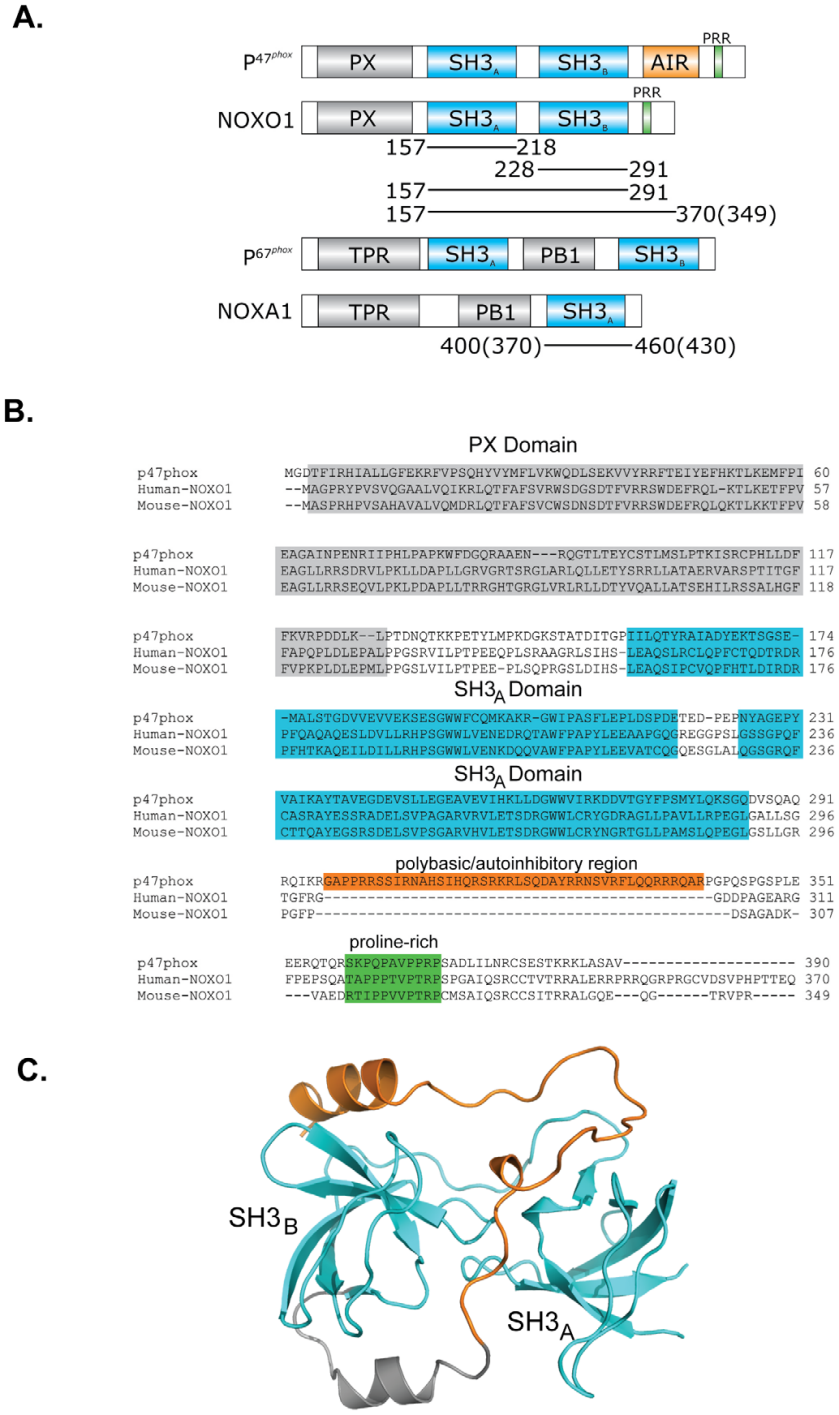
Schematic representation of the domain structures of p47^phox^, NOXO1, p67^phox^ and NOXA1. (**A**) Predicted domain structures of NOXO1 and NOXA1 in comparison to p47^phox^ and p67^phox^, respectively. Human and mouse constructs used in this study are illustrated by black lines. Mouse constructs are identical in length unless otherwise stated in brackets. The autoinhibitory region (AIR) and proline rich region (PPR) are indicated. (**B**) Alignment of p47^phox^, human NOXO1 and mouse NOXO1. The PX domains (grey shaded), SH3 domains (cyan shaded), polybasic region (orange shaded ox) and proline rich motif (green shaded) are indicated. (**C**) Structure of the autoinhibited core of p47^phox^ showing the superSH3 domain conformation. The structure shows the biologically relevant monomeric form of the protein that is also observed in solution, not the domain-swapped crystallized dimer [22]. The SH3 domains and polybasic region are highlighted in cyan and orange, respectively.

The phagocytic oxidase is still the best characterized member of this protein family and consists of a heterodimeric flavocytochrome (containing NOX2 and p22^phox^) that makes up the catalytic core of the enzyme plus the cytosolic regulatory subunits p40^phox^, p47^phox^ and p67^phox^ and the small GTPase Rac [8], [16]–[18]. Because excessive ROS production is cytotoxic and can induce a number of pathological processes, complicated regulatory mechanisms have evolved that tightly control the activity of the phagocytic oxidase. In the resting state, inappropriate activation is prevented through the partitioning of its subunits between the cytosol (p40^phox^, p47^phox^, p67^phox^ and Rac) and the membrane (NOX2, p22^phox^). The resting, cytoplasmic location of a trimeric p40-p67-p47^phox^ complex is maintained due to an autoinhibitory conformation of p47^phox^ that prevents the interaction with the membrane-bound flavocytochrome [18]. Cell activation induces phosphorylation of p47^phox^ at a number of serine residues leading to conformational changes and ultimately relieving autoinhibition. This in turn allows p47^phox^ to interact with a consensus PxxP motif within the cytoplasmic portion of p22^phox^, thereby promoting membrane translocation of the p40-p67-p47^phox^ complex and assembly of the active NADPH oxidase enzyme [19]–[22]. Due to these multiple activities of p47^phox^ this subunit plays a key role in NADPH oxidase function, acting simultaneously as a regulator of oxidase activity and an adaptor protein promoting enzyme assembly. In the autoinhibited state its tandem SH3 (Src homology 3) domains are engaged in an intramolecular interaction with a region rich in basic residues (polybasic region, also referred to as autoinhibitory region AIR) and hence are prevented from interacting with p22^phox^ [21], [23](Figure 1). The crystal structure of the autoinhibited core of p47^phox^ revealed that the tandem SH3 domains adopt an unusual, novel conformation, termed the superSH3 domain, in which the two SH3 domains co-operate to form a single ligand binding site that is occupied by a non-canonical motif located in the N-terminal portion of the polybasic region [22], [24]. Extensive, additional contacts are made between the polybasic region and the reminder of p47^phox^, outside of the core superSH3 domain ligand binding site that are important to enforce the resting state. Multiple serine residues within this polybasic region become phosphorylated upon activation, a process that weakens the intramolecular interactions and instead favours complex formation with the membrane-bound cytochrome. Additionally, a proline-rich region in the C-terminal portion of p47^phox^ mediates the interaction with p67^phox^ via specific recognition of its C-terminal SH3 domain. This interaction, which occurs with an unusually high affinity of 20 nM [25]–[27], is absolutely required for translocation of p67^phox^ to the membrane.

Although NOX1 was the first homologue of the phagocytic oxidase to be identified, its precise physiological function remains somewhat controversial although there is support for a role in host cell defense in the gastrointestinal tract and cell proliferation [11], [28]–[31]. For optimal activity NOX1 has been shown to require p22^phox^, Rac and two regulatory subunits, termed NOXO1 (NOX organizing protein 1) and NOXA1 (NOX activating protein 1) that have been identified based on their homology with p47^phox^ and p67^phox^, respectively [32]–[37]. The main difference between p47^phox^ and NOXO1 is the absence of the polybasic region that is important for autoinhibition and the regulation of the interaction with p22^phox^. NOXA1 contains a TPR (tetratricopeptide), PB1 (Phox and Bem1) and a carboxy-terminal SH3 domain, similar to p67^phox^, but lacks a centrally located SH3 domain (Figure 1).

The lack of the autoinhibitory region in NOXO1 combined with the observations that NOX1 produces superoxide constitutively at low levels and that a stimulus-induced increase in superoxide production only occurs in certain human cell types, has led to the suggestion that NOXO1 is permanently associated with p22^phox^ and that its activity is not regulated by phosphorylation or other signalling events that may induce changes in protein-protein interactions as seen for the phagocytic enzyme [32]–[34].

This study is aimed at providing the first quantitative description of the interactions taking place between NOX1 components in order to uncover the underlying mechanisms that are responsible for the observed stimulus-dependent increase in superoxide production. In particular we were interested in determining if the interaction between NOXO1 and p22^phox^ is indeed constitutive and if, in the absence of the autoinhibitory region, there is any need for the tandem SH3 domains to adopt a superSH3 domain conformation.

Our data clearly indicate that both SH3 domains of NOXO1 are required for the interaction with p22^phox^, strongly suggesting that it will form a superSH3 domain. Importantly though, only very weak binding was observed between p22^phox^ and a fragment of NOXO1 containing the tandem SH3 domains plus the C-terminal tail, implying that autoinhibitory interactions do indeed exist within NOXO1. Furthermore, unlike the phagocytic oxidase, complex formation between NOXO1 and NOXA1 is relatively weak, indicating that the molecular details of this interaction must be significantly different from those between p47^phox^ and p67^phox^. Finally we show that there are differences in the level of autoinhibition between the human and murine proteins, highlighting that the two systems might be regulated by different mechanisms.

## Results and Discussion

### NOXO1 forms a superSH3 domain that interacts tightly with p22^phox^

**Figure 2:**
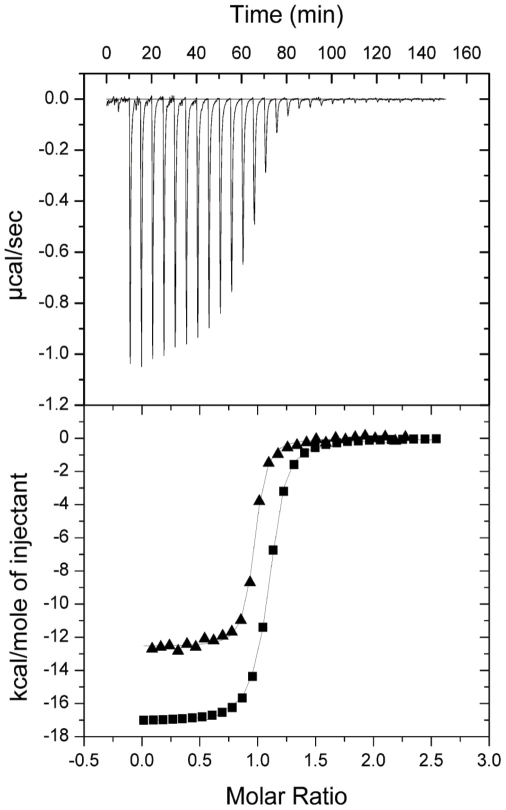
Characterisation of the interaction between the tandem SH3 domains of NOXO1 and peptides p22^phox^. Upper part shows the raw calorimetric data for the interaction of p22^phox^C and human NOXO1 SH3_AB_. Lower part shows the integrated heat changes, corrected for heat of dilution, and fitted to a single site binding model. (▪) Human p22^phox^C titrated into human NOXO1 SH3_AB_ (▴) Mouse p22^phox^C titrated into mouse NOXO1 SH3_AB_.

Our first aim was to establish if the tandem SH3 domains of NOXO1 co-operated in a manner similar to those of p47^phox^ and form a superSH3 domain that contains a flexible ligand binding site for the accommodation of different targets with high affinity and specificity [22]. Isothermal titration calorimetry (ITC) was used to measure binding of the individual human or mouse NOXO1 SH3_A_ and SH3_B_ domains to the p22^phox^ cytoplasmic domain. None of these titrations provided any evidence for complex formation, nor was there any binding between the isolated SH3 domains themselves (data not shown). This observation was confirmed using a fluorescently labelled p22^phox^-derived peptide and following changes in fluorescence intensity upon addition of increasing concentrations of human or mouse NOXO SH3_A_ and SH3_B_ domains. To ensure that lack of binding is not due to unfolding of the isolated SH3 domains, their structural integrity was confirmed by CD spectroscopy (data not shown). In contrast, the tandem SH3 domains of human and mouse NOXO1 bound tightly with affinities of 0.15 µM for human and 0.068 µM for mouse NOXO1 to the cytoplasmic domains (referred to as p22^phox^C) of human and murine p22^phox^, respectively (Table 1 and Figure 2). These data firmly establish that the tandem SH3 domains of NOXO1 need to co-operate to form a single unit that tightly interacts with p22^phox^ as previously observed in p47^phox^ (K_d_ p47-p22^phox^: 0.19 µM, [22]).

**Table 1:** Characterisation of intermolecular interactions between NOXO1 and p22^phox^.

Cell component	Syringe component	K_d_ (×10^−6^ M)	ΔH (kcal mole^−1^)		TΔS (kcal mole^−1^)
**Human NOXO1**					
SH3_AB_	p22^phox^C	0.15±0.01	−16.8±0.06		−7.7
SH3_AB–E_	p22^phox^C	17.5±1.0	−3.2±0.6		3.1
SH3_AB–E_ triple mutant	p22^phox^C	0.53±0.4	−8.1±0.5		−0.058
SH3_A_	p22^phox^C	NB			
SH3_B_	p22^phox^C	NB			
SH3_A_	SH3_B_	NB			
**Mouse NOXO1**					
SH3_AB_	p22^phox^C	0.068±0.01	−12.9±0.03		−3.3
SH3_AB–E_	p22^phox^C	0.78±0.06	−7.1±0.05		−0.29
SH3_A_	p22^phox^C	NB			
SH3_B_	p22^phox^C	NB			

All measurements were performed at 18°C. The *K*_d_ is given in units of 10^−6^ M, Δ*H* and TΔ*S* are given in kcal mol^−1^ (1 kcal/mol≡4.184 kJ/mol). The stoichiometry of complex formation for each binding site is N = 1.0±0.1. No binding is indicated by NB.

In light of this high-affinity interaction between the tandem SH3 domains of NOXO1 and p22^phox^ it is rather surprising that a NOXO1 fragment that lacks the PX (PhoX domain) domain is not capable anymore of co-localizing with NOX1 and p22^phox^ at the plasma membrane as described by Cheng and colleagues [38]. This observation implies that the high affinity for p22^phox^ must be diminished in the presence of additional sequences outside the tandem SH3 domains, possibly due to not yet identified autoinhibitory interactions.

### The presence of the C-terminal tail of NOXO1 significantly weakens the interaction with p22^phox^

**Figure 3:**
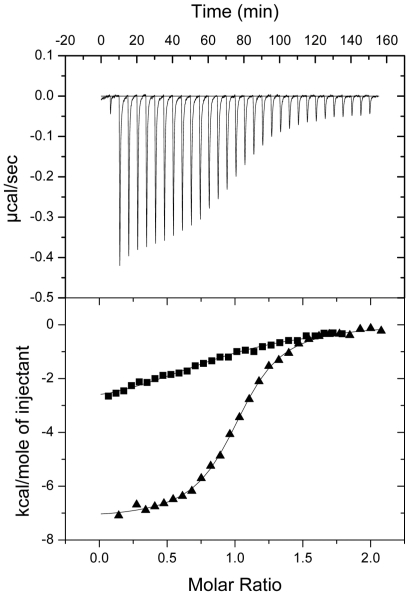
Characterisation of the interaction between NOXO1 SH3_AB–E_ and p22^phox^. Upper part shows the raw calorimetric data for the interaction of mouse p22^phox^C- NOXO1 SH3_AB–E_. Lower part shows the integrated heat changes, corrected for heat of dilution, and fitted to a single site binding model. (▪) Human p22^phox^C titrated into human NOXO1 SH3_AB–E_ (▴) Mouse p22^phox^C titrated into mouse NOXO1 SH3_AB–E_.

In the resting state, p47^phox^ is prevented from binding to p22^phox^ due to an intramolecular interaction between its tandem SH3 domains and the autoinhibitory region. This sequence is absent in human as well as mouse NOXO1 (Figure 1A and B). To determine if other motifs are present in the C-terminal tail of NOXO1 that may influence binding of the tandem SH3 domains to p22^phox^, ITC titrations were carried out with constructs harbouring the tandem SH3 domains plus the C-terminal portion of NOXO1 (referred to as SH3_AB–E_). Indeed, the interaction between human SH3_AB–E_ and p22^phox^ was weakened over 100-fold to 17.5 µM in comparison to the tandem SH3 domains (Figure 3). Interestingly, this inhibitory effect of the C-terminal tail of NOXO1 is less pronounced in the mouse protein. Nevertheless, binding to p22^phox^ is weakened by around 10-fold (K_d_ = 0.78 µM)(Figure 3 and Table 1). These results strongly suggest that autoinhibitory interactions do exist in both NOXO1 isoforms, but are more pronounced in the human protein. These data are particularly interesting in light of previous studies that showed that superoxide production by human NOX1 can be increased by stimulators such as PMA, while a similar increase could not be observed with the mouse proteins [32]–[34]. It is conceivable that the 0.78 µM tight interaction between murine NOXO1 and p22^phox^ is strong enough, even in the presence of inhibitory interactions with the C-terminal tail, to support full enzymatic activity, while the significantly weaker human complex (K_d_ = 17.5 µM) requires a conformational change to adopt a fully active NOXO1-p22^phox^ complex conformation.

Close examination of the C-terminal tail of human NOXO1 reveals a region comprising a number of basic residues that is absent in the mouse protein (see Figure 1B) (residues 347–359, ERRPRRQGRPRG). To test whether this region might contribute to the stronger autoinhibition in human NOXO1, a peptide corresponding to this region was synthesized and binding to the tandem SH3 domains was measured using ITC (data not shown). However, no binding was detected under the experimental conditions, suggesting that this is not the region responsible for the observed differences.

### The tandem SH3 domains of NOXO1 are involved in intramolecular interactions

SH3 domains classically bind to sequences harbouring a consensus PxxP motif. Such a motif is absent in the region responsible for autoinhibition in p47^phox^ but is present in the C-terminus of the protein. However, in the phagocytic enzyme this region mediates a very tight interaction with p67^phox^. A homologous motif is found in the C-terminal regions of human and mouse NOXO1 and is similarly believed to be the target of the SH3 domain of NOXA1 (Figure 1B)[34], [35]. To investigate if this region might be additionally involved in an intramolecular interaction with the tandem SH3 domains, a peptide corresponding to amino acids 319–347 of human NOXO1 (peptideA: TAPPPTVPTRPSPGAIQSRCCTVTRRALE) was titrated against the tandem SH3 domains as well as against the individual SH3_A_ and SH3_B_ domains of the human protein. A weak interaction with the tandem SH3 domains could be detected with a K_d_ of around 50 µM but not with any of the individual domains (data not shown). Interestingly this affinity is similar to that previously measured for the interaction between the isolated PX and SH3 domains in p47^phox^, which are believed to be engaged in an intramolecular interaction [39], consistent with a potential regulatory role. To gain further insight into the molecular details of this intramolecular interaction and to test the potential involvement of the conserved proline residues, which in the phagocytic enzyme are recognised by p67^phox^, we mutated proline residues 323, 326 and 329 to alanine in construct SH3_AB–E_ (human) and tested the ability of this mutant protein to interact with p22^phox^. Indeed, substitution of these proline residues strongly favoured the interaction with p22^phox^, which now occurred with almost the same affinity to that of the isolated tandem SH3 domains (K_d_ = 0.5 µM)(Table 1). The importance of this region for autoinhibition is further supported by the titration of the tandem SH3 domains of NOXO1 with a shorter version of peptideA, which only contains the proline rich region (TAPPPTVPTRPS) and was able to bind with a similar affinity as the long version of peptideA (K_d_∼50 µM). These results convincingly show that intramolecular interactions do exist in NOXO1, which include its tandem SH3 domains and the proline-rich sequence located C-terminally to it that interfere with the interaction with p22^phox^. Given the sequence conservation within the SH3 domains and proline-rich regions of human and mouse NOXO1 we do not understand at present why these autoinhibitory interactions are significantly more pronounced in human NOX1 and can only speculate that the length of the linker connecting the tandem SH3 domains and the proline-rich region, which is shorter in murine NOXO1, may be responsible for the observed differences.

### The interaction between NOXO1 and NOXA1 is weak

**Figure 4:**
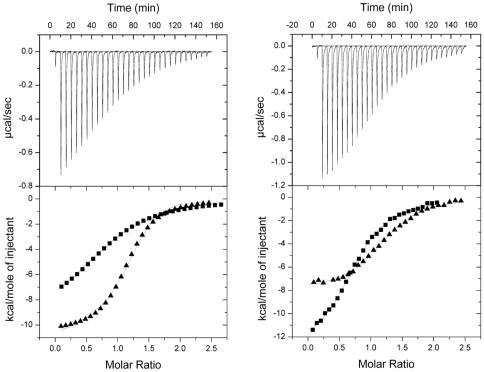
Characterisation of the interaction between NOXO1 and NOXA1. (**A**) Upper part shows the raw calorimetric data for the interaction between human NOXA1 SH3 and NOXO1 SH3_AB–E_. Lower part shows the integrated heat changes, corrected for heat of dilution, and fitted to a single site binding model. (▪) Human NOXA1 SH3 titrated into human NOXO1 SH3_AB–E_ (▴) Mouse NOXA1 SH3 titrated into mouse NOXO1 SH3_AB–E_. (**B**) Upper part shows raw calorimetric data for the interaction of peptideA and human NOXA1 SH3. Lower part shows the integrated heat changes, corrected for heat of dilution, and fitted to a single site binding model. (▪) PeptideA titrated into human NOXA1 SH3 (▴) PeptideA titrated into mouse NOXA1 SH3.

A previous study investigating the interaction between NOXO1 and NOXA1 showed that both proteins could be co-immunopricipitated from COS cells [34], which led to the suggestion that they interacted in a manner similar to p47^phox^ and p67^phox^. However, the data presented here clearly indicate that the proline-rich region of NOXO1 is engaged in an intramolecular interaction with its tandem SH3 domains and hence should not be available to NOXA1. To gain further insight into the molecular basis of complex formation between these two proteins we measured binding of the human and mouse NOXA1-SH3 domains to either NOXO1-SH3_AB–E_ construct by ITC. An interaction took place between the respective proteins, however, the complexes formed with >100-fold lower affinities (K_d_ = 7.2 µM for the human and K_d_ = 2.2 µM for the mouse proteins) than observed for their phagocytic counterpart (K_d_ = 20 nM, [26])(Figure 4A and Table 2). To test if this might be due to a competition between the tandem SH3 domains and NOXA1 for binding to the proline-rich motif we determined the affinity of either NOXA1-SH3 domain for peptideA (Figure 4B and table 2). Rather unexpectedly, the affinity of this peptide for NOXA1-SH3 (human: K_d_ = 5.8 µM, mouse: K_d_ = 5.9 µM) was not significantly different from the affinity of the SH3_AB–E_ construct, suggesting that the intramolecular interaction between the tandem SH3 domains and the proline-rich region has no influence on the interaction with NOXA1, which hence must recognize a different region. This conclusion is further supported by the observation that the short proline-rich motif-containing version of peptideA binds only with a very low affinity to NOXA1-SH3 (K_d_>70 µM; an exact determination of this dissociation constant was not possible due to a low heat change and the low affinity of the interaction). These observations are in contrast to a study by Yamamoto et al. who used GST pull-downs to investigate the NOXO1-NOXA1 interaction and found that wild-type NOXO1 failed to bind the SH3 domain of NOXA1, as suggested due to overlapping recognition motifs in the proline-rich region [40]. A present the reason for this discrepancy is not clear and we can only speculate that under the non-equilibrium conditions of pull-down assays one of the components may have been washed off.

**Table 2:** Characterisation of intermolecular interactions between NOXO1 and NOXA1.

Cell component	Syringe component	K (×10^−6^ M)	ΔH (kcal mole^−1^)	TΔS (kcal mole^−1^)
**Human**				
NOXO1 SH3_AB–E_	NOXA1 SH3	7.2±0.5	−8.6±0.7	−1.8
NOXA1 SH3	PeptideA	5.8±0.05	−12.6±0.6	−5.6
NOXA1 SH3	PeptideA-short	>70		
**Mouse**				
NOXO1 SH3_AB–E_	NOXA1 SH3	2.2±0.05	−10.1±0.8	−3.0
NOXA1 SH3	PeptideA	5.9±0.7	−7.6±0.3	−0.64

All measurements were performed at 18°C. The *K*_d_ is given in units of 10^−6^ M, Δ*H* and TΔ*S* are given in kcal mol^−1^ (1 kcal/mol≡4.184 kJ/mol). The stoichiometry of complex formation for each binding site is N = 1.0±0.1.

In the phagocytic oxidase the p47^phox^-p67^phox^ interaction is unusually strong (K_d_ of 20 nM) [25], [26], due to the presence of two protein interfaces: contacts are made via the conserved PxxPxR motif (at amino acids 363–368), but also via an additional surface composed of residues 369–390 of p47^phox^, which fold into a helix-turn-helix (HTH) motif and pack against β-sheets 2, 3 and 4 of SH3_B_ p67^phox^. Removal of this additional binding motif reduces the affinity for p67^phox^ to 20 µM. Based on the data presented in this manuscript, we now suggest that the interaction between NOXO1 and NOXA1 relies solely on this additional binding surface and does not include the proline-rich region. This scenario would be reminiscent of the behaviour of the SH3 domain of Pex13p, which binds to Pex14 in a conventional fashion via the recognition of a PxxP motif, while simultaneously interacting, via an independent surface, with a region in Pex15 that adopts an α-helical conformation [41], [42]. Our model is supported by the fact that the key residues of p47^phox^ in the HTH motif that make contact with p67^phox^ (I374 and T382) are conserved in human and murine NOXO1. Structural studies are now required to fully unravel the details of these interactions.

In this study we provide evidence that the assembly of the NOX1 oxidase complex can be regulated through reversible protein-protein interactions. We show that the region C-terminal to the tandem SH3 domains of NOXO1 interferes with binding to p22^phox^ and may thereby prevent the formation of a fully active oxidase complex in unstimulated cells. However, the inhibitory effect of the C-terminal tail is less pronounced in NOXO1 than in the homologous p47^phox^ subunit of the phagocytic oxidase, and may explain why the NOX1 system is capable of producing low levels of superoxide, in a constitutive manner. Based on available data we propose the following model for the regulation of NOX1 activity: NOXO1 is constitutively associated with NOX1 and p22^phox^ at the membrane, however, the complex with p22^phox^ can exist in a high and low affinity state. In the resting state a low basal level of superoxide production is required for cellular processes, and this activity is maintained by the low affinity complex between NOXO1 and p22^phox^ at the membrane. In this state the affinity of the tandem SH3 domains for p22^phox^ would be too low to allow a constitutive interaction with the membrane-bound cytochrome and the presence of the PX domain is required to ensure membrane localization of NOXO1, hence explaining the recent observation by Cheng and Lambeth that NOXO1 is cytosolic in the absence of the PX domain [38]. Upon activation with stimulators such as PMA, NOXO1 becomes fully active and allows a high affinity interaction with p22^phox^, thereby giving rise to an increase in the level of superoxide production. At present, it is unclear how cycling of NOXO1 between these two states is regulated and what the molecular details of this active state are, but our data suggest that full activation will require disruption of the intramolecular interaction between the tandem SH3 domains and the proline-rich region. In the case of the phagocytic oxidase, it is the phosphorylation of multiple serine residues in the polybasic region of p47^phox^ that induces these conformational changes. Equivalent phosphorylation sites are absent in NOXO1, suggesting that other mechanisms will regulate the interaction with p22^phox^. A recent report by Yamamoto et al. [40] showed that arachidonic acid could increase the interaction between NOXO1 and p22^phox^ and it was suggested that this lipid may be a physiological stimulator. Other scenarios that have not been investigated yet, such as changes in protein-protein interactions induced by phosphorylation of NOXA1 or p22^phox^ [43], [44] could also be envisaged and we cannot rule out the possibility of involvement of an additional unknown component. Further studies are now required to shed more light on the regulation of superoxide production in the NOX1 system.

## Materials and Methods

### Plasmid construction

Mouse NOXO1 and NOXA1 cDNA were a kind gift from Dr. B. Banfi (Geneva University Hospital, Geneva, Switzerland), human NOXO1α and NOXA1 from Dr. T. Leto (National Institutes of Health, USA) and human p22^phox^ was from Dr. M. C. Dinauer (Indiana University School of Medicine, Indiana, USA). Mouse p22^phox^ was obtained from Life Technologies Inc (I.M.AG.E CLONE Id: 5100892). The NOXO1, NOXA1, p22^phox^ full length proteins and fragments thereof were cloned into pGEX-6P1. Mutations were introduced by PCR-mediated site-directed mutagenesis using the Quikchange kit (Stratagene). All constructs were sequenced to confirm identities.

### Protein overexpression and purification

Proteins were expressed in *Escherichia coli* strain BL21 STAR (Invitrogen) (see Figure 1A for complete list of constructs). Cell cultures were induced at an optical density (*A*_600_) of 0.8 by the addition of 0.2 mM isopropyl β-D-thiogalactopyranoside and grown for an additional 3 hours at 28°C. The cells were harvested and the pellets were stored frozen at −70°C. Cell pellets were thawed in bufferA (50 mM HEPES, pH 7.4, 150 mM NaCl, 1mM EDTA, 1 mM DDT, with the addition of protease inhibitor tablets (Roche)). Cell membranes were disrupted by sonication on ice and supernatants obtained following a 1 hour centrifugation at 48,000×g. Recombinant GST-fusion proteins were purified by affinity chromatography on glutathione Sepharose 4B, washed with bufferA plus 500 mM NaCl and cleaved overnight in bufferA with PreScission Protease to remove the GST tag (Amersham Biosciences). Further purification was carried by gel filtration in bufferA on Superdex 75. The purity and identity of the proteins was confirmed by SDS/PAGE analysis (12 or 15% gels) and electrospray mass spectrometry. Synthetic peptides were prepared by P. Fletcher (NIMR) and Dr. W. J. Mawby (University of Bristol, UK).

### Isothermal titration calorimetry

Complex formation between NOXO1, NOXA1 and p22^phox^ fragments or peptides were measured by isothermal titration calorimetry using a MicroCal VP-ITC calorimeter (MicroCal Inc., Northampton, USA). All proteins were dialysed against ITC buffer (150 mM NaCl, 1 mM DTT and 50 mM HEPES, pH 7.4) and performed at 18°C. All peptides were dissolved in ITC buffer. Typically, 20–40 µM of protein in the cell was titrated by stepwise injections of a total of 200–300 µM of the ligand. Heats of dilution were determined by titrating protein or peptide into ITC buffer and subtracted from the raw ITC data prior to analysis with the software, Origin version 7.0, assuming a single-site binding model. Data were averaged over three to five experiments.

### Fluorescence Intensity Titration

Fluorescence titrations were carried out using an ISS PC1 spectrofluorometer. All measurements were carried out at 20°C with excitation at 492 nm and emission at 525 nm. A solution containing fluorescein labelled peptide1 (0.5 µM) was prepared in ITC buffer. Binding measurements were preformed by titrating increasing concentrations of NOXO1 SH3_A_ or SH3_B_ (1–50 µM) into this solution.
